# Role of the hippocampus in systems consolidation of remote fear memory

**DOI:** 10.1038/s12276-026-01680-9

**Published:** 2026-04-13

**Authors:** Hyunmin Park, Bong-Kiun Kaang

**Affiliations:** 1 Learning and Memory Group, Center for Memory and Glioscience, Institute for Basic Science, Daejeon, Republic of Korea; 2 Interdisciplinary Program in Neuroscience, Seoul National University, Seoul, Republic of Korea; 3 Department of Biological Sciences, Korea Advanced Institute of Science and Technology, Daejeon, Republic of Korea

**Keywords:** Consolidation, Hippocampus, Long-term memory

## Abstract

For decades, the processing and retrieval of memories over weeks or even years have been crucial and fundamental subjects in neuroscience. The hippocampus is essential for episodic memory, including associative fear memory. During remote fear memory formation, the hippocampus serves as an original source of fear memory and distributes memory contents to various brain regions, including the neocortex. Remote memory has a unique feature in terms of the cooperation between distributed regions to restore the original memory trace. Although the hippocampus has traditionally been considered crucial for recent memory processing, several studies have demonstrated that it is also directly involved in remote memory processing. This raises the crucial question: how does the hippocampus transfer the memory trace and at the same time retain recent memory properties? In this Review, we summarize various systems consolidation theories and compare the time-dependent changes in the hippocampus and neocortex. Subsequently, we address the role of the hippocampus and the molecular factors involved in remote memory processing, emphasizing the long-term engagement of the hippocampus. Finally, we aim to investigate memory transfer from the hippocampus to the cortical areas during systems consolidation and examine how this process contributes to fear memory generalization.

## Understanding remote fear memory and hippocampal function

The hippocampus is a key brain area for forming and recalling memories, including fear memories. Traditionally, scientists believed that the hippocampus only temporarily stores memories before transferring them to the neocortex for long-term storage. However, recent research challenges this view, suggesting the hippocampus remains involved in long-term memory processing. This review by researchers explores how the hippocampus interacts with other brain regions to support long-term memory. They discuss various theories, such as systems consolidation, multiple trace theory and index theory, which suggests ongoing hippocampal involvement in detailed memory recall. The authors highlight studies showing that, even after a long time, the hippocampus is active during memory recall and interacts with cortical areas such as the prefrontal cortex and anterior cingulate cortex. The findings suggest that the hippocampus plays a continuous role in memory retrieval and updating.

This summary was initially drafted using artificial intelligence, then revised and fact-checked by the author.

## Introduction

The hippocampus is well known as a critical region for the formation and retrieval of episodic memories, including fear memories. For decades, small populations of neurons known as engram neurons have been identified as key mediators of memory storage and retrieval^1–3^. The neocortex is essential for remote memory storage^4^. Rodent studies have shown that hippocampal engram cells are reactivated during recent memory recall, whereas neocortical engram cells are reactivated during remote memory recall^3,5,6^. This temporal reorganization between brain regions is considered to underlie remote memory formation, referred to as systems consolidation^7^. However, accumulating evidence supports the idea that the hippocampus plays an active role in remote memory processing. For instance, hippocampal lesion after learning impairs remote memory formation^8^, and inhibition of hippocampal subregions disrupts remote memory recall^9^. These findings challenge the classical concept of systems consolidation and raise the question of how and when the hippocampus transfers information to the cortical regions. In this Review, we discuss the development of systems consolidation theories and recent studies that support sustained hippocampal involvement in remote memory. In addition, we elaborate on the hippocampus-to-cortex circuits involved in this process and their implication for fear memory generalization.

## Main

### Systems consolidation theories

**Fig. 1:**
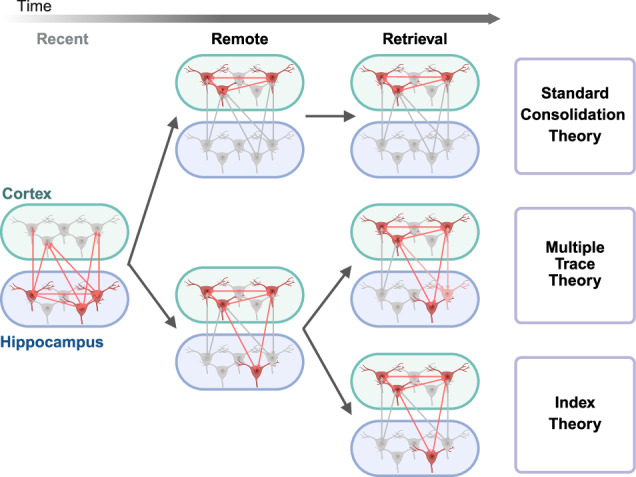
Systems consolidation theories focusing on the interaction between the hippocampus and neocortex. Top: SCT postulates that recent memory is dependent on the hippocampus, whereas remote memory becomes independent of it but instead acquires dependence on the neocortex over time. Middle: MTT shows the interdependence of the hippocampus and neocortex at a remote time point, with new hippocampal ensemble being recruited during remote retrieval. Bottom: IT represents the continuous interplay between the hippocampus and neocortex by maintaining coactivity between activated populations in both regions.

Memory consolidation is the stabilization process through which a newly formed memory is converted into a solid and enduring form. Memory consolidation includes synaptic consolidation and systems consolidation, which differ in mechanisms and temporal dynamics. Synaptic consolidation involves molecular and structural changes at individual synapses and neurons^10,11^, whereas systems consolidation is a process of memory storage reorganization throughout the distributed regions for long-term retention^12^. Various theories have sought to explain when and how the hippocampus interacts with cortical regions for remote memory formation, based on lesion studies and activity patterns in each region (Fig. 1).

The standard consolidation theory (SCT) represents a classical view of systems consolidation, in which memories are initially stored in the hippocampus and then transferred to the neocortex, forming a solid state of memory^7,13,14^. In the SCT, the hippocampus is regarded as a temporary memory system, whereas cortical regions are a storage for the permanent form of memory^15^. For instance, Rempel-Clower et al. reported that patients with bilateral hippocampal damage exhibited temporally graded retrograde and pronounced anterograde types of amnesia^16^. Likewise, patients with damage to the medial temporal lobe showed intact distant past and future memories, emphasizing the discrete roles of the hippocampus and cortex in memory storage based on temporal dynamics^17^.

Although the SCT accounts for the retention of remote memory following lesions in the hippocampus, accumulating evidence indicates that hippocampal damage also disrupts remote memory accuracy. In this regard, Nadel and Moscovitch argued that the SCT inadequately addresses this remote memory inaccuracy following lesions and subsequently proposed the multiple trace theory (MTT)^15^. According to the MTT, the hippocampus remains involved in detailed information, whereas the neocortex supports generalized remote memories, highlighting that continued interaction between these regions plays a crucial role in remote memory retrieval^18^. Building upon this MTT, trace transformation theory has been proposed to further account for the relationship between hippocampal lesions and retrograde amnesia. According to trace transformation theory, the hippocampus and distributed cortical regions encode distinct aspects of memory and engage in dynamic, bidirectional interactions throughout memory formation and retrieval^19^.

An intermediate explanation, the indexing theory (IT), is a modified version of MTT that adapts the SCT framework. Although remote memories are consolidated within cortical networks, a small population of hippocampal neurons retains an index that enables the retrieval of these distributed neocortical patterns^20^. Although the SCT conceptualizes the hippocampus and neocortex as functionally distinct storage systems, the IT suggests that integrated neocortical activity patterns reconstruct the original memory trace via the hippocampal index during remote retrieval. Supporting this explanation, O’Reilly and Rudy demonstrated that the hippocampus-associated entorhinal cortex could be the main cortical interface, suggesting that the entorhinal cortex forms hippocampus-modulated activity patterns. The findings support the IT in that the hippocampus plays a pivotal role in the retrieval of remote fear memory through its dynamic interaction with cortical regions encoding consolidated memory traces^21^. Collectively, these theories highlight the continuing involvement of the hippocampus in remote memories and that the hippocampus and neocortex are interdependent during remote memory formation and retrieval.

### Hippocampus for remote fear memory

During systems consolidation, memory is gradually processed in distributed brain regions and becomes less dependent on the hippocampus^12,22^. Multiple studies have suggested that the hippocampus contributes to memory formation and subsequent reorganization in a temporally limited manner. For instance, hippocampal lesions more than 7 days after contextual fear conditioning did not result in impaired behavior in rats, suggesting a dissociation between the hippocampus integrity and memory retention^23,24^.

However, accumulating evidence indicates that the hippocampus also plays a role in the formation and retrieval of remote fear memories. Lesion studies have shown that damage in the hippocampus impairs both short-term and long-term memories in rats^25,26^. At the subregional level, optogenetic engram reactivation in the dentate gyrus (DG) induces freezing behavior in infant mice^27^, and optogenetic manipulation of DG engram neurons affects 2-week memory retrieval^5,28^. Similarly, using CA3-TeTx mice, disruption of CA3 during memory acquisition blocks the consolidation of fear memory and intrinsic oscillation within the hippocampus, leading to impairment of remote memory^8^. By contrast, optogenetic inhibition of the CA1 impairs very remote memory persisting more than 6 months^9^. Collectively, these studies suggest that distinct hippocampal subregions are essential for remote memory formation. The hippocampus is actively engaged in remote memory recall. Several studies have demonstrated that the hippocampus is reactivated during remote retrieval, indicating that it can store information and engage in memory retrieval for up to 14 days after learning^29,30^. A functional connectome study further revealed that, whereas the absolute hippocampal activity decreases over time, the connectivity of the hippocampus with other brain regions is strengthened, which is associated with remote memory^31^. In addition, hippocampal oscillatory coupling is important for remote memory recall. For memories older than 7 days, anterior cingulate cortex (ACC) theta oscillation modulates the CA1 gamma activity, exhibiting phase-locked activity that is related to context information^32,33^. Furthermore, during the recall of 4-week remote memory, the coupling of amygdala gamma oscillation and hippocampal theta oscillation becomes more synchronized and refined, even as the overall activity became weaker^34^. Together, these findings highlight that hippocampal engram cells retain the original memory trace and modulate signals to cortical regions for remote memory retrieval and systems consolidation.

Memory traces can be reactivated and become hippocampus dependent again, a process referred to as systems reconsolidation. During systems reconsolidation, inhibition of protein synthesis in the hippocampus using anisomycin disrupts the reconsolidation of memory older than 4 weeks^35,36^. During systems reconsolidation, the reactivated hippocampal trace is thought to provide a scaffold for integrating new information^37^. For example, rats with hippocampus lesions showed higher freezing in a generalized context, even when the reminder triggered reconsolidation of specific fear memory, indicating that the hippocampus is essential for remote memory reconsolidation^38^. Recent findings further demonstrated that the hippocampus recruits new engram cells through new proteins synthesis and restabilizes updated memory representation via interaction with the amygdala^39^. In addition to the dorsal hippocampus, ventral hippocampal CA1 also interacts with basolateral amygdala and plays a crucial role in remote reconsolidation^40^. Collectively, these studies suggest that the hippocampus is important for remote reconsolidation through interacting with the amygdala.

### Associated molecules in the hippocampus for remote memory

**Fig. 2:**
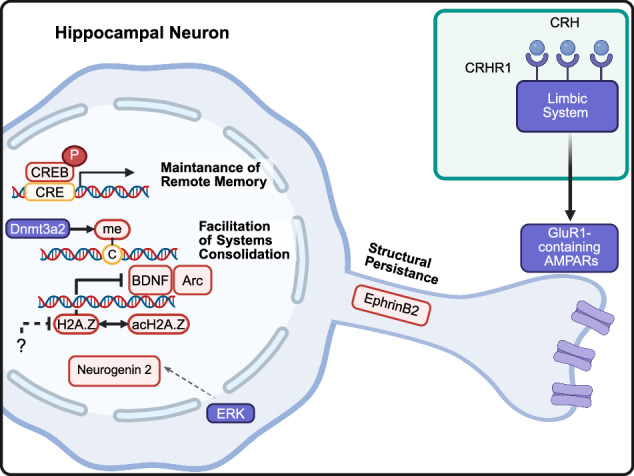
Remote memory-associated molecules in the hippocampus. In the nucleus, the transcription factor pCREB supports the maintenance of remote memory after memory acquisition. In addition, histone modifications such as histone acetylation and methylation facilitate systems consolidation and improve remote fear memory. ERK5 and EphrineB2 support structural changes after memory formation and Ca^2+^ permeable AMPARs are crucial for sustaining remote memories in the hippocampus. P, phosphorylation; me, methylation.

Although the molecular mechanisms underlying recent memory encoding in the hippocampus, such as the calcium-calmodulin-dependent kinase II pathway^41^, are well established, relatively few studies have investigated the molecular substrates that contribute to remote memory formation. However, recent studies have begun to identify molecular substrates related to signaling pathways, structural plasticity and epigenetic modifications associated with remote memory formation (Fig. 2).

One extensively studied molecule is cAMP response element-binding protein (CREB), which is a transcription factor essential for long-term potentiation. Following fear retrieval after 50 days in rats, phosphorylated CREB (pCREB) levels increase in the hippocampus, correlating with the freezing ratio, suggesting that the CREB pathway contributes not only to the formation of recent memory but also to the maintenance of remote memory^42^. In addition, corticotropin-releasing hormone receptor 1 (CRHR1) in the limbic system is involved in the consolidation of remote fear memories, modulating hippocampal protein expression. Specifically, limbic CRHR1 facilitates remote memory consolidation by promoting the expression and stabilization of the GluR1-containing AMPA receptors in the DG. Twenty-eight days after learning, treatment of CRHR1 antagonist induced the decreased GluR1 expression in the DG, which is closely correlated with changes in remote freezing behavior^1^. These studies suggest that the signaling pathways that are involved in recent memory encoding may also be involved in remote memory processes.

Furthermore, structural plasticity within the hippocampus is associated with remote memory formation^43^. EphrinB2, a molecule that regulates spinogenesis, is upregulated shortly after learning. EphrinB2 is increased in CA1 shortly after learning, and this might affect the cortical engram formation, impairing the ACC synaptogenesis for remote memory formation^44^. Similarly, extracellular signal-regulated kinase 5 (ERK5), essential for adult neurogenesis, is involved in both hippocampal neurogenesis and memory formation^45^. Conditional ERK5 knockout after memory acquisition disrupted remote but not short-term or recent memory formation. Furthermore, ERK5 knockout before memory acquisition, as well as 1 and even 5 weeks after acquisition, impaired remote fear memory, indicating that ERK5 is critically involved in both memory formation and long-term maintenance of memory^46^. In addition to signaling and structural mechanisms, epigenetic modifications have emerged as essential regulators of remote memory in the hippocampus^47^. For instance, 30 min after memory acquisition, histone variant H2A.Z binding around the transcription start site becomes altered, which consequently affects the expression of immediate-early genes (IEGs). Depletion of H2A.Z in the CA1 region enhanced both 1-day and 2-week fear memory, which was accompanied by changes in IEG expression patterns^48^. Moreover, blocking of H2A.Z through Tip60 inhibition improves 7-day fear memory^49^. These studies suggest that histone H2A.Z modification might regulate both recent and remote memories, with distinct modification patterns contributing to each memory phase. Furthermore, overexpression of the DNA methyltransferase Dnmt3a2 in the hippocampus enhanced the activation of the ACC neurons, and inhibition of these neurons impaired 2-week memory, suggesting that DNA methylation within the hippocampus influences cortical activity involved in remote memory maintanance^50^. Collectively, these findings suggest that epigenetic modification may influence the hippocampal structural plasticity, enabling a subset of engram neurons to retain the original memory trace.

### Hippocampus to cortex for systems consolidation

Interaction between the hippocampus and other brain regions is a fundamental process of systems consolidation that underlies remote memory formation. Regarding fear memory, the hippocampus primarily interacts with the neocortex, amygdala and thalamus. This section focuses on the interaction between the hippocampus and neocortex during systems consolidation.

#### Hippocampus to mPFC

The medial prefrontal cortex (mPFC) plays a central role in encoding and retrieval of remote memory^51–53^. Lesions in the mPFC impair remote fear retrieval in rats^24^ and activity within this region increases during remote, but not extinguished, fear memory retrieval^6,54^. During remote memory formation, the hippocampus transfers the memory trace to the mPFC. Based on the c-Fos activity, network connectivity analyses suggested that hippocampal activity correlates with mPFC activity during remote memory retrieval^54^. Consistent with this finding, functional ultrasound imaging has demonstrated that the hippocampus is functionally connected to the prelimbic cortex, indicating that this connectivity correlates with the behavioral expression of fear^55^.

Anatomically, the hippocampus directly projects axons to the mPFC^56^, and this circuit is crucial for remote memory expression^57^. Through the modulation of this pathway, hippocampal activity influences mPFC function, thereby contributing to systems consolidation. For instance, lesions in the mPFC and hippocampus induced impaired fear memory in rats^58^. Within the ventral CA1–mPFC circuit, hippocampal neurons projecting to the mPFC are preferentially activated during context exposure and are thought to modulate the transfer of the fear memory trace^57,59^. Taken together, these findings indicate that the interaction between the hippocampus and mPFC is a key candidate for systems consolidation, which progressively transfers memory from the hippocampus to the mPFC over time.

#### Hippocampus to ACC

As in the case of interactions with the mPFC, hippocampal activity modulates the ACC function to support remote memory formation. During remote memory retrieval, the ACC plays a dominant role but remains on the hippocampal input for at least 21 days^60,61^. Several studies have suggested that hippocampal engram neurons alter their activity levels and projections to the ACC. Specifically, following memory formation, the number of CA1 axons projecting to the ACC increases over time as memories mature^43^. In addition, astrocytes within CA1 contribute to remote memory formation^62^ and facilitate axonal recruitment toward the ACC^63^, suggesting the possibility that hippocampal astrocytes might facilitate the recruitment of ACC-projecting CA1 neurons, thereby contributing to remote memory formation. Consistently, hippocampal lesions attenuate ACC spinogenesis following remote memory consolidation, supporting an interdependent role of the hippocampus and ACC during systems consolidation^44,64^.

Furthermore, interactions between the hippocampus and ACC are regulated by oscillation coupling during sleep. ACC activity is mediated by CA1 activity, which may represent an underlying mechanism of memory transfer^65^. Ripple–spindle coupling during sleep is enhanced after learning, and the disruption of this coupling hinders both recent and remote fear memory^66^. Notably, the DG engram neurons, which regulate CA3 pattern separation, enhance the reactivation of ACC engram neurons during 2-week memory retrieval^67^. These findings highlight the time- and hippocampal activity-dependent recruitment of the ACC during systems consolidation.

#### Hippocampus to RSC

The retrosplenial cortex (RSC) serves as a hub for sensory integration and spatial memory. RSC is increasingly regarded as a crucial region for fear memory formation and retrieval^2,68^. RSC engages in remote memory processing in a manner different from that of other cortical regions. Although the inactivation of CA1 attenuated the fear memory, the reactivation of RSC alone was sufficient to retrieve fear memory which is independent of hippocampal representation^69^. Nevertheless, RSC activity can be reinforced by the hippocampal engram cells, thereby contributing to remote memory consolidation^70,71^.

The RSC is involved not only in recent^72,73^ but also in remote fear memory^74^. Optogenetic activation of the RSC reproduced features of remote memory, such as enhanced neocortical connections and reduced hippocampal dependence^75^. These studies suggest that RSC functions as a mediator for remote memory formation, facilitating the distribution of memory traces processed within the hippocampus. Supporting this hypothesis, the inhibition of the hippocampus-to-RSC circuit during memory acquisition impaired remote memory formation^70,76^. The dorsal CA1–RSC circuit is crucial for cortical engram maturation. Ablation of engram neurons in either CA1 or RSC disrupts remote memory formation and impairs mPFC engram maturation, suggesting that the CA1–RSC pathway conveys critical memory content required for systems consolidation^77^. Furthermore, the theta band in the RSC showed increased coherence with CA1 local field potentials during context exposure and fear retrieval^72^, and this oscillatory coordination might support systems consolidation^78^. In addition, an increase in the hippocampus-to-RSC interaction during phasic rapid eye movement sleep is associated with memory retention^79^. These studies have shown that the hippocampus orchestrates RSC activity both directly and indirectly, which is essential for the long-term stabilization and retrieval of fear memories.

### Fear generalization in the hippocampus

Fear generalization refers to the phenomenon in which conditioned fear responses are expressed not only in the original conditioned context but also in novel or similar contexts^80^. This generalization can be adaptive, enhancing survival by promoting caution in uncertain environments, but excessive generalization has been implicated in anxiety disorders such as post-traumatic stress disorder in humans^81^. The generalization of fear memories is considered to be the result of the distribution of memory traces from the hippocampus to cortical regions during systems consolidation, rather than restriction to the intrahippocampal circuits^82^.

During the initial stages of memory formation, the hippocampus plays a central role in encoding precise contextual information through pattern separation. Impairment of this pattern separation increases fear generalization within 10 days, highlighting that distinct engram patterns in the hippocampus are key modulators of precise fear memory^28,83,84^. As memory undergoes systems consolidation, memory dependence on the hippocampus is reduced, whereas the cortical regions become progressively actively involved in fear memory retrieval^61,85^. Under the systems consolidation and transformation hypothesis, contextual information becomes less precise over time as memory becomes increasingly independent of the hippocampus.

In particular, IEG expression levels in the hippocampus are reduced in the generalized context, suggesting that decreased discrimination is associated with reduced hippocampal activity^86^. In parallel, the ventral hippocampus has been implicated in the modulation of fear generalization at remote time points. The chemogenetic inhibition of the ventral hippocampus reduces freezing in a novel context, in cooperation with the ACC, which assumes a more prominent role in memory retrieval in the consolidated state^30,87^. Collectively, these findings suggest that fear generalization is a consequence of the distribution of memory traces during systems consolidation. This distribution process involves dynamic interactions between the hippocampus and various cortical regions, including the ACC. This implies that its role at remote time points may differ from its well-known function in recent memory encoding, which involves participation in memory state after the distribution and transformation of memory.

## Discussion

In this Review, we discussed the role of the hippocampus in remote memory formation, its interaction with other cortical regions, and the association between generalized fear memory and systems consolidation. The hippocampus is not merely a temporal region involved in recent memory processing but also serves as a critical output hub, transferring memory traces via engram neurons to other brain regions. During systems consolidation, the hippocampus primarily interacts with various cortical regions to establish and stabilize remote fear memories. Notably, the hippocampus continues to actively engage in fear retrieval for more than 2 weeks, indicating that the hippocampus preserves the original memory representation over extended periods alongside its cortical representation. Memory traces are distributed in various cortical regions, which may result in the loss of precision and generalized fear memories. However, extended involvement of the hippocampus in remote memory retrieval remains controversial. Although earlier studies reported that the hippocampus did not show higher activity during remote retrieval^6^, more recent studies have demonstrated the higher hippocampal activity and persistent reactivation of hippocampal engram neurons during remote memory retrieval^29,43^. However, there are relatively few mechanistic studies in which hippocampal activity is documented to modulate interactions with other brain regions during systems consolidation. In addition, the time points classified as ‘remote’ vary substantially across studies, which may contribute to confusion regarding the hippocampal contribution to systems consolidation at recent, intermediate and remote stages.

Focusing on hippocampal activity, hippocampal engram neurons may alter the transfer of the memory trace to the cortex, in a way such that dependence on the hippocampus during systems consolidation becomes lower, but the properties of the original memory trace are retained. Further research is needed to elucidate how information is transferred from the hippocampus to various cortical regions and how these interactions contribute to memory precision and generalization. In addition to interregional coordination, hippocampal engram neurons may alter their features during systems consolidation via the underlying molecular mechanisms, thereby reducing the dependency of the original memory trace on the hippocampus over time. Finally, it may be crucial to investigate the coordinating activity in various brain regions via the hippocampus. Several studies have suggested that many brain regions, such as the amygdala and thalamus, are involved in systems consolidation^88,89^. Future studies need to address how the hippocampus modulates and integrates information transfer across the distributed multiple regions and how this mechanism of transfer occurs, in relation to neuronal activity coupling between the hippocampus and various brain regions.

In terms of synaptic plasticity, connectivity among engram neurons is essential for memory formation and maintenance in the hippocampus and amygdala, which reflects memory states^90,91^. Moreover, synaptonuclear signaling, which refers to retrograde signaling from activated synapse to the nucleus, plays a critical role in synaptic potentiation^92,93^ and induction of gene expression in the nucleus^94,95^. In the context of remote memory, mechanistic studies have predominantly focused on the cellular and circuit-level modifications, whereas how individual synapses linking the hippocampus with downstream postsynaptic regions are persistently modified over time remains poorly understood. Future studies that address synaptic mechanism underlying remote memory will be essential to elucidate how remote memories are encoded and maintained through long-lasting synaptic modification.
